# Causes of death and predictors of childhood mortality in Rwanda: a matched case-control study using verbal social autopsy

**DOI:** 10.1186/s12889-018-6282-z

**Published:** 2018-12-17

**Authors:** Neil Gupta, Lisa R. Hirschhorn, Felix C. Rwabukwisi, Peter Drobac, Felix Sayinzoga, Cathy Mugeni, Fulgence Nkikabahizi, Tatien Bucyana, Hema Magge, Daniel M. Kagabo, Evrard Nahimana, Dominique Rouleau, Amelia VanderZanden, Megan Murray, Cheryl Amoroso

**Affiliations:** 1 Division of Global Health Equity, Brigham & Women’s Hospital, Boston, USA; 2 Northwestern University Feinberg School of Medicine, Chicago, USA; 3 Partners In Health/Inshuti Mu Buzima, Rwinkwavu, Rwanda; 4 Department of Global Health and Social Medicine, Harvard Medical School, Boston, USA; 5 Ministry of Health of Rwanda, Kigali, Rwanda; 6 Ariadne Labs, Boston, USA

## Abstract

**Background:**

Rwanda has dramatically reduced child mortality, but the causes and sociodemographic drivers for mortality are poorly understood.

**Methods:**

We conducted a matched case-control study of all children who died before 5 years of age in eastern Rwanda between 1st March 2013 and 28th February 2014 to identify causes and risk factors for death. We identified deaths at the facility level and via a community health worker reporting system. We used verbal social autopsy to interview caregivers of deceased children and controls matched by area and age. We used InterVA4 to determine probable causes of death and cause-specific mortality fractions, and utilized conditional logistic regression to identify clinical, family, and household risk factors for death.

**Results:**

We identified 618 deaths including 174 (28.2%) in neonates and 444 (71.8%) in non-neonates. The most commonly identified causes of death were pneumonia, birth asphyxia, and meningitis among neonates and malaria, acute respiratory infections, and HIV/AIDS-related death among non-neonates. Among neonates, 54 (31.0%) deaths occurred at home and for non-neonates 242 (54.5%) deaths occurred at home. Factors associated with neonatal death included home birth (aOR: 2.0; 95% CI: 1.4–2.8), multiple gestation (aOR: 2.1; 95% CI: 1.3–3.5), both parents deceased (aOR: 4.7; 95% CI: 1.5–15.3), mothers non-use of family planning (aOR: 0.8; 95% CI: 0.6–1.0), lack of accompanying person (aOR: 1.6; 95% CI: 1.1–2.1), and a caregiver who assessed the medical services they received as moderate to poor (aOR: 1.5; 95% CI: 1.2–1.9). Factors associated with non-neonatal deaths included multiple gestation (aOR: 2.8; 95% CI: 1.7–4.8), lack of adequate vaccinations (aOR: 1.7; 95% CI: 1.2–2.3), household size (aOR: 1.2; 95% CI: 1.0–1.4), maternal education levels (aOR: 1.9; 95% CI: 1.2–3.1), mothers non-use of family planning (aOR: 1.6; 95% CI: 1.4–1.8), and lack of household electricity (aOR: 1.4; 95% CI: 1.0–1.8).

**Conclusion:**

In the context of rapidly declining childhood mortality in Rwanda and increased access to health care, we found a large proportion of remaining deaths occur at home, with home deliveries still representing a significant risk factor for neonatal death. The major causes of death at a population level remain largely avoidable communicable diseases. Household characteristics associated with death included well-established socioeconomic and care-seeking risk factors.

## Background

The Millennium Development Goal #4 to reduce child mortality by two thirds by the end of 2015 resulted in an unprecedented focus on child health and a 52% reduction in sub-Saharan Africa between 1990 to 2015, from 179 to 86 deaths per 1000 live births [1]. Despite global achievements, only one-third of the priority countries in sub-Saharan Africa reached their child mortality target. This achievement gap translated into an estimated 2.9 million child deaths in 2015 in sub-Saharan Africa, of which two-thirds were likely from preventable causes [2] and 45% occurred in the neonatal period. Poverty and maternal education continue to play a key role in determining child mortality, and severe challenges remain in health care financing, human resources, service utilization, and information systems [3–5].

Rwanda, a country of 11.4 million people in the Great Lakes region of Africa, has made dramatic improvements in child mortality, moving from 37 neonatal and 152 under-5 deaths per 1000 live births in 2005 to 20 and 50 per 1000 live births, respectively, in 2015 [6]. This reduction in child mortality occurred in the context of multiple cross-sectional national and decentralized interventions designed to increase access to care, improve health resources, and strengthen the provision of effective care. These included the national introduction of a community health insurance program, high coverage for vaccination and vitamin A supplementation, implementation of community-based Integrated Management of Childhood Illness (IMCI), provision of insecticide treated nets, near elimination of mother to child HIV infections, increase in facility-based deliveries, and mobile phone reporting/support for emergency pre- and post-natal care [7–9].

Despite these efforts, child mortality in Rwanda, as in other countries in resource-poor regions of sub-Saharan Africa, varies widely across the country. Child mortality remains higher in the Eastern Province of Rwanda, which has the highest infant (51 deaths per 1000 live births) and under-5 mortality (86 deaths per 1000 live births) as compared to other provinces [6]. Nationally, under-5 mortality is largely associated with poverty (84/1000 live births in the lowest wealth quintile vs. 40/1000 live births in the highest quintile), maternal education (89/1000 live births in mothers with no education vs. 43/1000 for women with secondary education or higher), and residence (70/1000 in rural areas vs. 51/1000 in urban areas) [7]. However, the immediate causes and sociodemographic drivers for mortality particularly among those in the lowest wealth quintiles are poorly understood.

In order to fill this knowledge gap, we implemented a verbal social autopsy (VSA). In addition to the biologic factors identified in traditional verbal autopsy interviews, VSA allows for in-depth understanding of social, behavioral, and systems determinants of childhood death, as well as the caregiver’s experience and perspectives, thereby identifying potential modifiable targets in the home, community, and health system [10–12]. We conducted a matched case-control study utilizing an enhanced VSA interview tool to determine probable cause of and predictive factors for childhood deaths in an area of high rates of childhood mortality in Rwanda.

## Methods

### Study setting

This study was conducted in two rural Eastern Province hospital catchment areas covering approximately 529,000 individuals. In this intervention area, the Ministry of Health (MOH) facilities have been financially and technically supported by the non-governmental organization Partners In Health/Inshuti Mu Buzima (PIH/IMB) since 2005.

In Rwanda, the health system includes three main levels: community, health center, and district hospital. Community health workers (CHWs) provide household level health education, case finding for acute and chronic illness, community IMCI (including diagnosis and treatment of pneumonia, diarrhea, and malaria), female contraception, and linkage to health facilities for prenatal care, deliveries, and other medical services [13]. Each of the 23 health centers serve a catchment area of approximately 20,000–30,000 people and are staffed by general nurses who provide basic diagnostics, outpatient acute services, family planning, prenatal care, and routine deliveries. The average walking distance from households to the nearest health facility is estimated at just over an hour in Kayonza and over an hour and a half in Kirehe [14]. Reflecting national standards, district hospitals in Eastern Province are staffed by general practitioners and nurses who provide secondary care for advanced or inpatient care for patients referred from health centers, including comprehensive obstetric emergencies requiring cesarean section, neonatal care, and inpatient treatment for severe childhood illness and severe malnutrition.

### Study design and data collection

We conducted a matched case-control study of all children who died before 5 years of age in the study area between 1st March 2013 and 28th February 2014. We identified deaths using multiple sources including facility registers, community health worker reports, monthly review of CHW-held community death records, and a database from a mobile phone-based reporting system, the Monitoring of Vital Events using Information Technology (MoVe-IT), which was introduced in these two districts in 2012 to improve vital events reporting [15].

After confirming childhood deaths with local CHWs, we conducted interviews with caregivers of the deceased child in their households. Trained data collectors approached caregivers between three weeks to one year following the child’s death. Each case was matched to two controls selected from the nearest households with a child in a comparable age group (for neonates, children aged 1–30 days; for infants, children aged 31 days up to 1 year; and for children older than one year, matched to those between 1 and 5 years) to the deceased child. Neonatal cases without an available control under 30 days of age were matched with infants up to 180 days of age. Prior to the VSA, interviewers asked families of neonatal deaths additional questions in order to screen out potential cases of stillbirth. We obtained written informed consent from the caregivers of the deceased children and those selected as controls. The current caregiver of the child was not necessarily the biological mother if the biological mother was not available or was deceased. Using a questionnaire based on the 2012 World Health Organization verbal autopsy (VA) tool [16] supplemented with questions from the Rwanda MOH’s Under-5 Death Audit Tool and the 2010 Rwanda Demographic and Health Survey, we obtained information on the case or control child’s demographic characteristics, information on the child’s birth, illness, care seeking, and the family’s perceptions of care.

### Analysis

We used InterVA4 [17] to determine probable causes of death and cause-specific mortality fractions (CSMF) for each cause of death. The InterVA algorithm uses a range of health indicators taken from interviews as input and applies Bayes’ Theorem to determine the likeliest cause of death. The CSMF is an output from the algorithm and can be interpreted as the total number of deaths attributable to a specific cause. Prevalence of HIV and malaria were entered as “high” in the InterVA model, based on national level facility reporting indicating an estimate of greater than one in 100 deaths due to each of these diseases [18]. We estimated odds ratios for a range of child, caretaker, household, and care-seeking characteristics using conditional logistic regression. We retained variables with *p*-value less than or equal to 0.2 significance level in the univariate analyses in a multivariate model. We performed multiple imputations to infer values of missing data, which were considered missing completely at random. We used a bidirectional elimination stepwise method, which uses both forward selection and backward elimination in succession to determine optimal variables, to arrive at a final model in which the remaining variables were significant at the α = 0.05 level. Risk factors with potential collinearity were not included in multivariate analysis. We analyzed deaths in neonates (day of life 0 to 28) and non-neonates (day of life 29 to 5 years) separately. We used Global Burden of Disease level 1 categories [19] to organize causes of death by communicable, maternal, neonatal, and nutritional disorders (Group 1), non-communicable diseases (Group 2), and injuries (Group 3).

### Ethical approval

This study was approved by the Rwanda National Ethics Committee and Partners Institutional Review Board under the Population Health Implementation and Training program, a partnership between PIH/IMB, the University of Rwanda, and the Rwanda MOH. All caregivers who participated provided informed consent and were informed that they were able to discontinue participation at any time during the interview.

## Results

### Mortality results

Overall, 618 deaths were identified during the study period: 174 (28.2%) in neonates and 444 (71.8%) in non-neonates. For neonates, the median age of death was 2 days (interquartile range [IQR]: 0–7); 49.4% occurred in the first 48 h and 75.9% in the first week of life (Table 1). Nearly one-third (32.8%) of neonates did not visit a hospital or health center during the course of illness; 39.9% of neonates received no treatment for the illness that preceded their death. Among those that did seek care prior to the death, 16.7% were first evaluated by a community health worker; more than half were first evaluated either at a health center (43.7%) or district hospital (12.6%). Of the 174 neonatal deaths, data regarding discharge were present for 127 neonates. Of those, 97 (76.4%) were never discharged from the hospital following birth. Sixteen percent of the neonates were born through non-facility delivery. One hundred and eight (62.1%) of the neonatal deaths were in health facilities and 54 (31.0%) were at home. The majority (CSMF 66.7%) of identified causes of neonatal death were from Group 1 communicable or neonatal causes including pneumonia and birth asphyxia. The CSMF for deaths from Group 2 (non-communicable diseases) was 2.0%; 31.3% could not be assigned a specific cause (Table 2).

**Table 1:** Characteristics of under-5 deaths

	All U5 (*N* = 618) *N* (%)	Neonate (*N* = 174) *N* (%)	Non-Neonate (*N* = 444) *N* (%)
Sex (*n* = 618)			
Male	347 (56.2)	104 (59.8)	243 (54.7)
Female	271 (43.9)	70 (40.2)	201 (45.3)
Timing of neonatal death (*n* = 170)			
Died within 24 h of birth	–	69 (40.6)	–
Died between 24 and 48 h of life	–	15 (8.8)	–
Died between 48 h and 1 week of life	–	45 (26.5)	–
Died between 1 week and 28 days of life	–	41 (24.1)	–
First type of care sought (*n* = 618)			
Traditional healer	50 (8.1)	8 (4.6)	42 (9.4)
Community health worker	193 (31.2)	29 (16.7)	164 (36.9)
Health center	209 (33.8)	76 (43.7)	133 (29.9)
District hospital	31 (5.0)	22 (12.6)	9 (2.0)
Private pharmacy	5 (1.0)	0 (0.0)	5 (1.1)
Other	19 (3.1)	4 (2.3)	15 (3.4)
Unknown	32 (5.2)	12 (6.9)	20 (4.5)
No care sought	79 (12.8)	23 (13.2)	56 (12.6)
Attended health facility (hospital or health center) during illness (*n* = 616)			
No	202 (32.7)	57 (32.8)	145 (32.7)
Yes	416 (67.3)	117 (67.2)	299 (67.3)
Received any treatment at a health facility for the illness that led to death (*n* = 608)			
No	197 (32.4)	67 (39.9)	130 (29.5)
Yes	411 (67.6)	101 (60.1)	310 (70.5)
Place of death (*n* = 618)			
Hospital	156 (25.2)	80 (46.0)	76 (17.1)
Health center	74 (12.0)	28 (16.1)	46 (10.4)
Home	296 (47.9)	54 (31.0)	242 (54.5)
Other	91 (14.7)	12 (6.9)	79 (17.8)
Unknown	1 (0.2)	0 (0.0)	1 (0.2)
Discharged from health facility following birth (*n* = 127)			
No (died in health facility)	–	97 (76.4)	–
Yes	–	30 (23.6)	–

**Table 2:** Cause-specific mortality fractions for neonates (day of life 0 to 28) and non-neonates (day of life 29 to 5 years)

Age group	GBD Group 1 (Communicable, maternal, neonatal, and nutritional disorders)		GBD Group 2 (Non-communicable diseases)		GBD Group 3 (Injuries)	
	Cause of death	CSMF	Cause of death	CSMF	Cause of death	CSMF
Neonates	Neonatal pneumonia	26.5	Congenital malformation	1.0		
	Birth asphyxia	11.3	Epilepsy	0.6		
	Other and unspecified neonatal COD	8.5	Other and unspecified cardiac disease	0.4		
	Meningitis and encephalitis	7.9				
	Prematurity	6.4				
	Neonatal sepsis	5.6				
	Acute abdomen^a^	0.5				
	TOTAL	66.7	TOTAL	2.0	TOTAL	–
Non-neonates	Malaria	34.7	Epilepsy	2.3	Accidental drowning and submersion	1.3
	Acute resp. infections, including pneumonia	19.7	Congenital malformation	0.9	Road traffic accident	0.7
	HIV/AIDS-related death	12.0	Asthma	0.7	Other and unspecified external COD	0.6
	Acute abdomen^a^	7.6			Other transport accident	0.6
	Diarrheal diseases	5.9			Assault	0.5
	Meningitis and encephalitis	1.2				
	Other and unspecified infect disease	0.9				
	Severe malnutrition	0.9				
	TOTAL	82.9	TOTAL	3.9	TOTAL	3.7

31.3% of neonatal deaths and 9.5% of non-neonatal deaths identified as “indeterminate”

*COD* cause of death, *CSMF* Cause-specific mortality fraction, *GBD* Global Burden of Disease

^a^May have mixed etiologies

Among non-neonates, just under one-third (32.7%) of cases did not visit a hospital or health center during the course of illness; 29.5% received no treatment for the illness that preceded their death (Table 1). Among those that did seek care prior to the death, 36.9% were first evaluated by a community health worker; about one in three were first evaluated either at a health center (29.9%) or district hospital (2.0%). The median age of death was 515 days (IQR: 192–998); 122 deaths (27.5%) were in health facilities and 242 (54.5%) were at home. More than three-quarters of these deaths (CSMF 82.9%) were classified as due to Group 1 diseases including malaria, acute respiratory infections, HIV/AIDS related deaths, and diarrheal diseases (Table 2). Other avoidable causes included Group 2 diseases (CSMF 3.9%) and Group 3 causes (injuries) (CSMF 3.7%); 9.5% deaths could not be assigned a specific cause.

### Risk factors for death among neonates

In the univariate analysis, we identified the following as risk factors for neonatal death: female child (OR: 0.8; 95% CI: 0.7–1.0), home birth (OR: 1.8; 95% CI: 1.3–2.7), multiple gestation birth (OR: 1.9; 95% CI: 1.1–3.3), both parents deceased (OR: 3.8; 95% CI: 1.2–12.7), non-use of family planning methods (OR: 0.8; 95% CI: 0.6–1.0), and barriers to care including lack of accompaniment to a health facility (OR: 1.5; 95% CI: 1.1–2.1) and caregiver assessment of services received as moderate to poor (OR: 1.5; 95% CI: 1.1–2.0) (Table 3). In multivariate analysis, neonates who died were more likely than controls to have been born at home (aOR: 2.0; 95% CI: 1.4–2.8), born as part of a multiple gestation (aOR: 2.1; 95% CI: 1.3–3.5), and to have both parents deceased (aOR: 4.7; 95% CI: 1.5–15.3). They were less likely to have a mother who reported using family planning methods (aOR: 0.8; 95% CI: 0.6–1.0), and were more likely to have a caregiver who reported that not having a family member available to accompany them to a health facility was a barrier to care (aOR: 1.6; 95% CI: 1.1–2.1), and who assessed the medical services they received as moderate to poor (aOR: 1.5; 95% CI: 1.2–1.9).

**Table 3:** Risk factors for neonatal death as compared to matched controls

Univariate Analysis					Multivariate Analysis			
Child, Caretaker, or Household Characteristic	Odds Ratio	95% Confidence Interval		*p*-value	Odds ratio	95% Confidence Interval		*p*-value
Female child	0.8	0.7	1.0	0.091	0.8	0.7	1.0	0.084
Mother with primary education or less	0.9	0.6	1.4	0.655	–	–	–	–
Mother age ≤ 18	1.4	0.5	3.9	0.506	–	–	–	–
Mother age > 35	0.8	0.4	1.6	0.564	–	–	–	–
Mother with health insurance	0.8	0.6	1.2	0.340	–	–	–	–
Both parents deceased	3.8	1.2	12.7	0.027	4.7	1.5	15.3	0.01
Household size ≥5	1.2	0.9	1.5	0.206	–	–	–	–
No electricity in the house	0.9	0.5	1.6	0.744	–	–	–	–
Natural/rudimentary walls in the house	0.8	0.6	1.2	0.310	–	–	–	–
No mosquito nets in the house	1.0	0.7	1.5	0.962	–	–	–	–
Mother not using family planning	0.8	0.6	1.0	0.041	0.8	0.6	1.0	0.042
Home delivery	1.8	1.3	2.7	0.002	2.0	1.4	2.8	0.000
Multiple gestation delivery	1.9	1.1	3.3	0.016	2.1	1.3	3.5	0.005
Mother with ≤1 antenatal care visit	1.3	0.9	1.8	0.233	–	–	–	–
Caretaker reported financial barrier to care	1.1	0.9	1.5	0.372	–	–	–	–
Caretaker reported seeking permission as a barrier to care	1.0	0.5	1.8	0.991	–	–	–	–
Caretaker reported distance to health facility as a barrier to care	1.0	0.7	1.4	0.946	–	–	–	–
Caretaker reported not having an accompanying person as a barrier to care	1.5	1.1	2.1	0.015	1.6	1.1	2.1	0.007
Caretaker reported a poor to moderate perception of health facility services	1.5	1.1	2.0	0.004	1.5	1.2	1.9	0.002

### Non-neonates case-control results

In the univariate analyses, children who died were more likely than controls to have been a part of a multiple gestation birth (OR: 2.8; 95% CI: 1.7–4.7), not to have received adequate vaccinations (OR: 1.7; 95% CI: 1.2–2.3), and to come from a larger household (OR: 1.2; 95% CI: 1.0–1.4). Mothers of non-neonatal cases were more likely than age-matched controls to have primary education or less (OR: 1.9; 95% CI: 1.2–3.0) and not use family planning methods (OR: 1.6; 95% CI: 1.4–1.8). Caregivers were less likely to have health insurance (OR: 0.9; 95% CI: 0.7–1.0) and more likely to report no household electricity (OR: 1.4; 95% CI: 1.0–1.8) as well as financial barriers to care (OR: 1.1; 95% CI: 1.0–1.3) (Table 4). In the multivariate model, children who died were more likely than controls to have been born as part of a multiple gestation (aOR: 2.8; 95% CI: 1.7–4.8), to not have received all scheduled vaccinations (aOR: 1.7; 95% CI: 1.2–2.3), and to come from a large household (aOR: 1.2; 95% CI: 1.0–1.4). Caretakers were also more likely to report lower maternal education levels (aOR: 1.9; 95% CI: 1.2–3.1), mothers not using family planning (aOR: 1.6; 95% CI: 1.4–1.8), and electricity lacking in the household (aOR: 1.4; 95% CI: 1.0–1.8). The variable “mother health insurance” was not included in the multivariate analysis due to potential collinearity with “caretaker reported financial barrier to care”.

**Table 4:** Risk factors for non-neonatal death as compared to matched controls

Univariate Analysis					Multivariate Analysis			
Child, Caretaker, or Household Characteristic	Odds Ratio	95% Confidence Interval		*p*-value	Odds ratio	95% Confidence Interval		*p*-value
Female child	0.9	0.8	1.1	0.241	–	–	–	–
Mother with primary education or less	1.9	1.2	3.0	0.006	1.9	1.2	3.1	0.005
Mother age ≤ 18	0.8	0.4	1.7	0.554	–	–	–	–
Mother age > 35	1.3	0.8	2.0	0.235	–	–	–	–
Mother with health insurance	0.9	0.7	1.0	0.111	–	–	–	–
Both parents deceased	1.2	0.7	1.8	0.490	–	–	–	–
Household size ≥5	1.2	1.0	1.4	0.029	1.2	1.0	1.4	0.037
No electricity in the house	1.4	1.0	1.8	0.052	1.4	1.0	1.8	0.041
Natural/rudimentary walls in the house	0.9	0.8	1.2	0.566	–	–	–	–
No mosquito nets in the house	0.9	0.7	1.2	0.475	–	–	–	–
Mother not using family planning	1.6	1.4	1.8	<.0001	1.6	1.4	1.8	<.0001
Multiple gestation delivery	2.8	1.7	4.7	<.0001	2.8	1.7	4.8	<.0001
Child not adequately vaccinated	1.7	1.2	2.3	0.003	1.7	1.2	2.3	0.003
Caretaker reported financial barrier to care	1.1	1.0	1.3	0.155	1.1	0.8	1.3	0.109
Caretaker reported seeking permission as a barrier to care	0.9	0.7	1.3	0.714	–	–	–	–
Caretaker reported distance to health facility as a barrier to care	1.0	0.8	1.1	0.649	–	–	–	–
Caretaker reported not having an accompanying person as a barrier to care	1.1	0.9	1.3	0.482	–	–	–	–
Caretaker reported a poor to moderate perception of health facility services	1.0	0.9	1.2	0.962	–	–	–	–

## Discussion

In the context of rapidly declining childhood mortality in Rwanda and increased access to and utilization of formal health care, we found a large proportion of remaining deaths occur at home, and home deliveries still representing a significant risk factor for neonatal death. The major causes of death at a population level remain largely avoidable communicable diseases. Household characteristics associated with death included well-established socioeconomic and care-seeking risk factors. To our knowledge, this is the first study to explore the causes of death in Rwanda using household-level VSA, including a case-control design to also explore the association of socio-demographic and access factors with the risk of neonatal and childhood deaths.

Although the proportion of neonatal deaths in the first week of life in this cohort (75.9%) was similar to previous reports based on vital registration data, [20, 21] our results showed a lower proportion of neonatal asphyxia and prematurity-related deaths than both regional estimates and previous results in Rwanda [22, 23]. These findings may reflect the contribution of programs in these districts targeting survival of premature and full-term infants through improved neonatal resuscitation techniques and immediate newborn care, including introduction of continuous positive airway pressure at the hospital level [24]. For non-neonatal deaths, the causes of death estimated from our study continue to attribute a high overall cause of death to preventable communicable disease, driven in large part by malaria, pneumonia, and diarrheal diseases. Despite national malaria control efforts, there are higher rates of malaria incidence in the Eastern region of Rwanda [25]; in addition, the country experienced an upsurge in cases of malaria during the time of data collection, attributed to climate changes, pyrethroid resistance, sub-standard insecticide-treated bednets, and inconsistent application of indoor residual spraying [18, 26]. The relative proportion of deaths attributable to pneumonia was consistent with both regional and historical rates [21, 23]. In contrast, a lower proportion of deaths were attributable to diarrhea than previously reported [23], likely due in part to the expansion of community IMCI and efforts to improve quality of facility-based IMCI [27].

In multivariate analysis, home birth was a significant risk for neonatal mortality, and 16% of cases of neonatal death identified in this study occurred in neonates with nonfacilitydelivery. This supports findings among cohorts in Uganda [28] and Indonesia [29] which showed substantially elevated mortality risk associated with birth at home. This increased risk may be due to the lack of immediate access to emergency clinical obstetric or neonatal care among home births without skilled attendants as well as low utilization of post-partum care and services [30]. Orphanhood, or both parents deceased, was also associated with higher odds of death among neonates, and has been reported elsewhere to be a major risk factor for childhood mortality [31]. A previous study in Tanzania found that children whose mothers died during the early maternal period had a 50% chance of surviving to one year of age compared to a 94% probability among children with living mothers [32].

Several established risk factors were confirmed in our research, including multiple gestation birth [31, 33], lower maternal education [4, 34, 35], larger household size [34, 36], absence of family planning use [37, 38], and incomplete vaccination [39]. Lack of household electricity was another significant factor for non-neonatal deaths, which may be directly associated with greater indoor air pollution from other energy sources [40], an increase in injuries and accidents related to solid fuel use [41], or be a surrogate for other household vulnerabilities. Household electricity may also be associated with greater family or community resources that provide a protective effect on under-5 health outcomes [42, 43].

Self-reported barriers to accessing care were significantly associated with deaths in the neonatal age group. Lack of a family member or other person to accompany a caregiver to the health facility was independently associated with neonatal death, which likely resulted in delays in care seeking and may also be reflected in the higher risk of death associated with home birth.

Lack of insurance was not significantly associated with deaths in either age category in the analysis. Given the high rates of insurance coverage for mothers in cases and controls, our study may have lacked sufficient power to detect significant risk factors for death. Whereas a community-based health insurance scheme has been associated with increased utilization of child services at health facilities in Rwanda [44], user fees still exist in the form of co-payment, which may to some degree still limit access to care [45], findings supported from initial qualitative analysis of the VSA interviews [46]. Inadequate utilization of family planning, which was independently associated with childhood deaths in this study, may reflect lower health seeking-behaviors or poorer access to care by caretakers of children who died compared to caretakers of the control children.

Our study had a number of limitations. While the updated 2012 WHO VA tool and InterVA4 algorithm in use are confirmed to have high reliability compared to other vital case reporting standards [47, 48], the accuracy of the identified cause of death from the algorithm in this country and context is unknown, especially for neonates, where a large proportion of deaths were categorized as indeterminate cause, or for particular conditions, such as malnutrition, which may go under-detected using this methodology. Additionally, VSA relies on caregiver recall of events surrounding the death, which introduces issues related to caregiver memory of the events and responder bias, particularly given the sensitivity of an event as significant as the death of a child. In particular, this bias may influence assessment of barriers to care. The InterVA4 algorithm requires prevalence assumptions for the rate of deaths attributed to HIV in the background population. According to national facility-based reporting, 1% of childhood deaths and 6% of all deaths in 2014 were associated with HIV/AIDS, though this may have overestimated the proportion of overall childhood deaths related to HIV/AIDS, where a rapidly declining HIV vertical transmission rate and improving diagnostics and access to treatment have dramatically reduced HIV/AIDS-related death in the pediatric population in Rwanda [49]. Additionally, while efforts were made to collect data from multiple new and existing data sources, some deaths may have been missed, particularly deaths initially thought to be stillbirths in both facility and community settings, thereby potentially underestimating the proportion of neonatal deaths. Childhood vaccination coverage was not corrected for survival, and therefore it is possible that the relationship between complete vaccination coverage and childhood mortality may have been overestimated due to the additional time that passed between the death date of the case and the interview date for the control, during which the control may have received additional vaccinations. Despite standardized procedures to collect interview data, data is missing for several variables; however, we believe these data are missing at random, and do not bias our findings. Finally, while we had caregiver report of perceived quality, we do not know the technical quality of care provided and the relative contribution to amenable deaths.

## Conclusion

Despite significant health system improvements and rapid declines in childhood mortality in Rwanda, a large proportion of child deaths in this study occurred at home. While there was a high proportion of facility-based deaths for neonates, home deliveries still represent a major risk factor for neonatal death. Significant financial and care-seeking barriers remain with a high proportion of deaths occurring outside of the health system for non-neonates, which present clear targets for focused interventions at both the community and health facility levels. Despite overall gains in childhood mortality, well informed policies, guidelines, and system improvements targeting key risk factors identified in this study could accelerate gains for neonatal and childhood survival in rural Rwanda and similar contexts across sub-Saharan Africa.

## Abbreviations

CHW: Community health worker
CSMF: Cause-specific mortality fraction
GBD: Global Burden of Disease
IMB: Inshuti Mu Buzima
IMCI: Integrated Management of Childhood Illness
MOH: Ministry of Health
MoVe-IT: Monitoring of Vital Events using Information Technology
PIH: Partners In Health
VA: Verbal autopsy
VSA: Verbal social autopsy
WHO: World Health Organization
